# The primary cilium as a multifunctional organelle: emerging roles and unanswered questions

**DOI:** 10.1186/s12964-025-02403-9

**Published:** 2025-10-02

**Authors:** Denis Corbeil, Kristina Thamm, Jana Karbanová, Christine A. Fargeas, József Jászai

**Affiliations:** 1 Biotechnology Center (BIOTEC) and Center for Molecular and Cellular Bioengineering, Technische Universität Dresden, Tatzberg 47-49, Dresden, 01307 Germany; 2 Tissue Engineering Laboratories, Medizinische Fakultät der Technischen Universität Dresden, Fetscherstr. 74, Dresden, 01307 Germany; 3 Institute of Anatomy, Medizinische Fakultät der Technischen Universität Dresden, Fiedlerstr. 42, Dresden, 01307 Germany; 4 Tissue Engineering Laboratories, Biotechnology Center, Technische Universität Dresden, Tatzberg 47-49, Dresden, 01307 Germany; 5 Present Address: BIONCaRT GmbH, Fürstenweg 8, Grimma, D-04668 Germany

**Keywords:** Adhesion, CD133, Cilium, Extracellular vesicle, Intercellular communication, Prominin-1, Signal transduction, Microtubule

## Abstract

The primary cilium, a solitary membrane-bound, microtubule-based cellular organelle, has been considered an evolutionary relict for almost a century. Over the past three decades, interest in this protruding, non-motile structure of the plasma membrane has been boosted by the identification of ciliary dysfunctions as the underlying cause of developmental abnormalities and inherited disorders, commonly called ciliopathies. The primary cilium responds to environmental stimuli, such as mechanical, chemical, or light (in the case of the modified cilium of photoreceptors) signals. The membrane of primary cilia host specific sensory complexes and/or receptors associated with various pathways, predisposing them to transmit (or convert) spatiotemporal environmental information into cellular response. These dual mechanochemical aspects led to the recognition that primary cilia are multifunctional sensory organelles that act as “cellular antennae”. Beyond their established role in signal transduction, primary cilia are newly recognized as important hubs for short- and long-distance intercellular communication due to their ability to release and, perhaps, selectively take up extracellular vesicles, which are biological carriers exchanged between cells. In addition, the physical contact of the primary cilium with other cilia, cytonemes or with nerve cell axons adds another layer of complexity to the mechanisms of sensory and/or intercellular communication between neighboring cells that needs to be further explored. In this review, we focus on these new and less-explored ciliary properties and processes, which can affect cell communication and signaling and thus have a direct impact on development, tissue homeostasis, and pathological conditions.

## Introduction

Cilia, hair-like structures protruding several micrometers from the cell surface, are associated with a broad spectrum of eukaryotic cells ranging from unicellular entities (algae and protozoa) to complex tissues of vertebrates. Cilia are indispensable for normogenesis, tissue repair and adult homeostasis [1, 2].

**Fig. 1:**
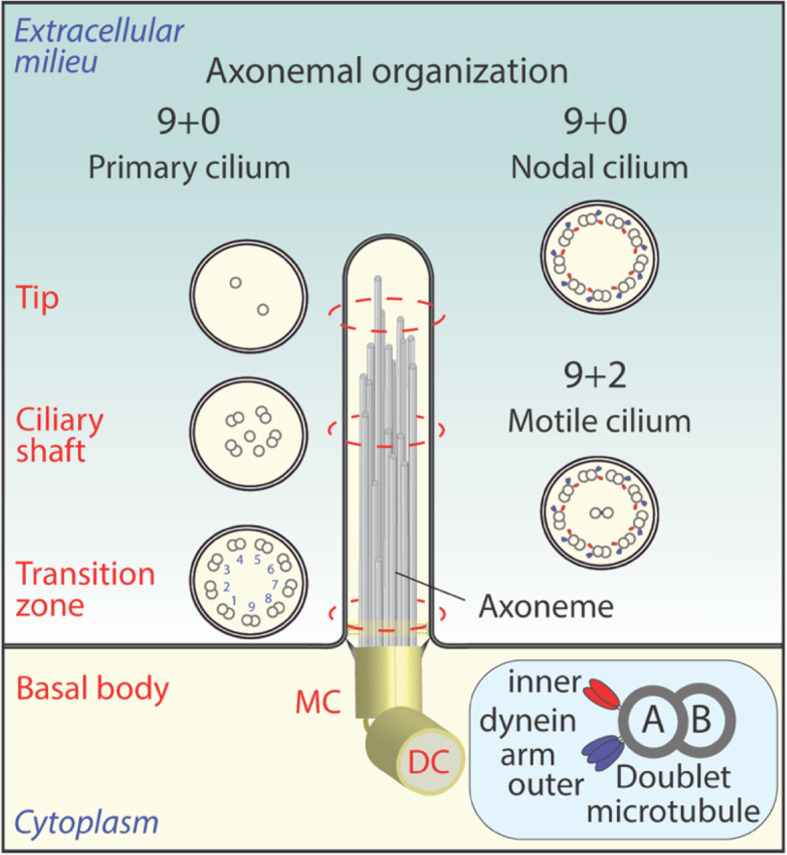
Axonemal architecture of the non-motile primary cilium in comparison to nodal or other motile cilia. Transverse sections showing the arrangement of the axoneme through the primary cilium (left), nodal cilia and other motile cilium (right). Nine peripheral doublet microtubules are present in all ciliary structures differing by the absence (9 + 0 arrangement) or presence (9 + 2 arrangement) of the central pair of singlet microtubules located at the core of the axoneme. Note that the axoneme extending from basal body has variable configurations along the primary cilium, from its base (transition zone) to its tip, ending in singlet microtubules. Doublet microtubules (A- and B-tubules, see inset) and the inner and outer dynein arms responsible for ciliary motility are shown. The latter are absent in the non-motile primary cilium. Items are not drawn to scale. DC, daughter centriole; MC, mother centriole. Illustrations are based on data and/or schemes presented in Refs [9–17]

The common core to all ciliary structures is the axoneme, a microtubular internal framework. Nine axonemal microtubule doublets extend from the basal body, a modified centriole with 9 microtubule triplets, and run in a precise circumferential array along the longitudinal axis of cilia. The axonemal microtubule doublets consist of an A and a B tubule (Fig. 1). Cilia that lack a central pair of microtubules (9 + 0 arrangement) were classified as non-motile solitary cilia or primary cilia. The latter were first considered to be those with sensory functions (see below). Variations in the axonemal architecture have resulted in different types of cilia, and perhaps also distinct functions. Motile cilia, in contrast to primary cilia, have a central pair of singlet microtubules (9 + 2 arrangement) that enable their motility (Fig. 1). The functional relationship of the 9 + 2 arrangement with ciliary motility has been extensively documented, particularly in connection with another related but highly evolved ciliary structure: the sperm flagellum. This long, specialized, archetypal ciliary structure is responsible for propelling the sperm cell by rhythmic beating [3]. Likewise, the planar and well-orchestrated beating of motile cilia found either on the multiciliated epithelia of the respiratory tract or on the ependymal cells of the cerebral ventricles is the driving force behind mucociliary clearance and cerebrospinal fluid circulation, respectively [4]. However, the discovery of cilia with an axonemal structure (9 + 2), typically found in motile cilia, fulfilling sensory functions has challenged the traditional classification of cilia, necessitating a more inclusive taxonomy [5–8].

Structurally, the situation is further complicated by the existence of cilia with a special 9 + 4 axonemal arrangement [18], or of primary cilia, where the 9 + 0 symmetry pattern observed at the base of the cilium is broken along the ciliary length ending in individual microtubules (Fig. 1) [6, 9–13] (see Commentaries in Refs [14, 15] and references therein). Adding to the complexity of the ciliary structures and functions, it became evident that motility is not a feature unique to those with a 9 + 2 axonemal configuration. For example, pit cells of the embryonic node, the left-right organizer in early vertebrate embryogenesis, are equipped with 9 + 0 monocilia that are motile (Fig. 1). Clockwise circular beating of the central nodal cilia generates a leftward flow of extracellular fluid, containing vesicular parcels and potentially other factors, which are sensed by the more peripheral immotile mechanosensitive cilia. This triggers an asymmetric calcium signaling, which is essential for activating the downstream signaling cascades that determine, at the molecular level, the left-right axis preceding organogenesis [19–25] (reviewed in Refs [26, 27]. Moreover, the specialized cilia, called kinocilia, which are found either transiently on the hair cells of the inner ear in the mammalian auditory system or permanently on those of the vestibular organ, are considered as non-motile, although they embody an axoneme with a 9 + 2 arrangement. Yet, their passive deflection could contribute to sensing of sound waves as well as spatial orientation and movement of the head, respectively. Similarly, numerous non-motile cilia with a 9 + 2 arrangement emanate from the apical dendritic knob of olfactory sensory neurons and are associated with specific chemosensory functions (see Refs [28–30] and references therein).

**Fig. 2:**
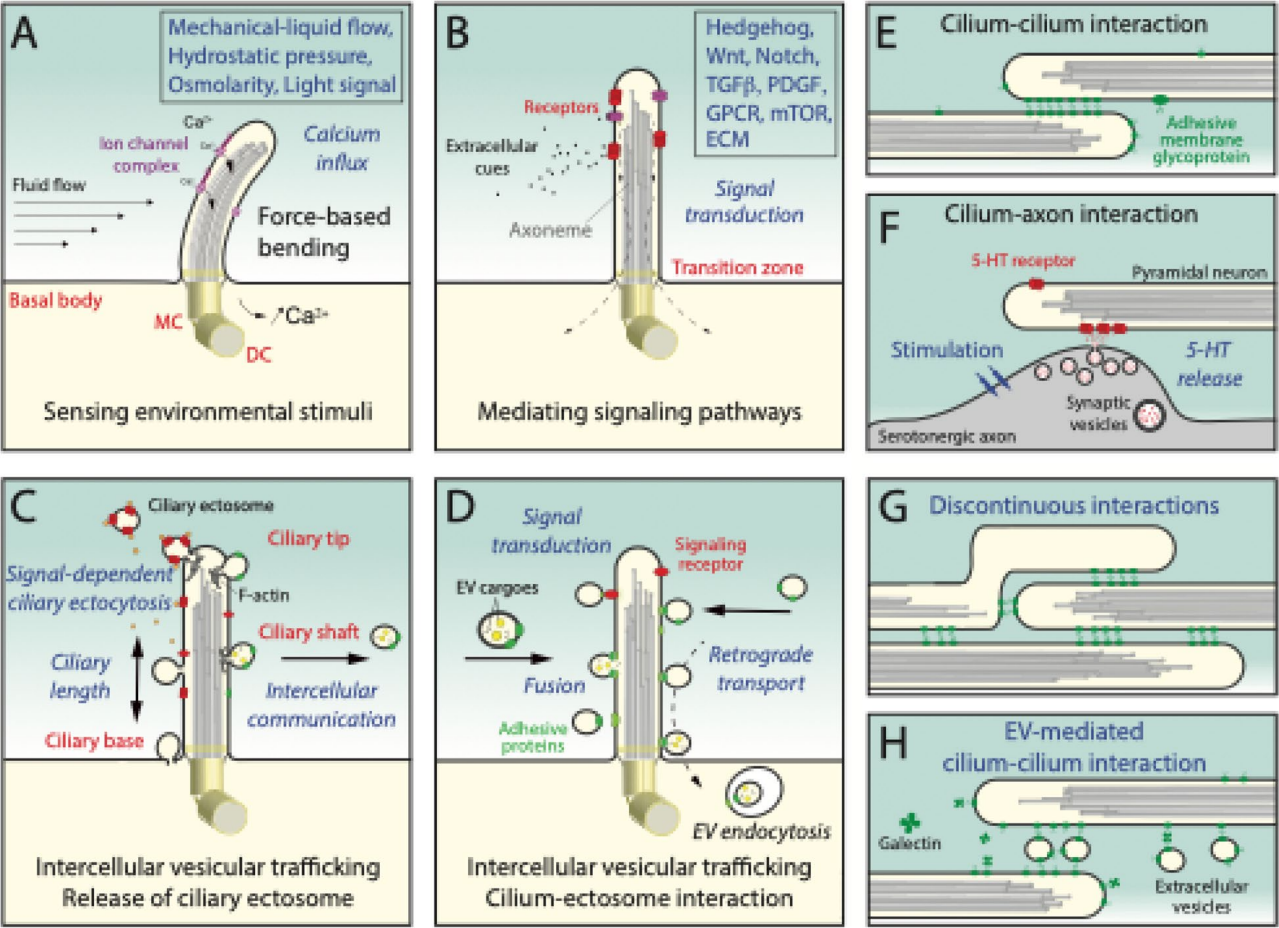
The primary cilium – a multifunctional organelle implicated in various cellular processes. **A**, **B** Primary cilium senses environmental stimuli like mechanical forces (fluid shear stress) or other signals (**A**) as well as biochemical cues that lead to the activation of various signal transduction pathways (**B**) and intracellular responses. **C**, **D** Primary cilia are the source of ciliary ectosomes released either from the ciliary tip, shaft, or possibly from the ciliary base or its immediate surrounding areas (**C**). Primary cilia can serve as EV docking sites (**D**), playing a dual role in intercellular vesicular trafficking. After docking, EVs can move towards the ciliary base, where they can potentially be endocytosed, delivering their cargoes to receptor cells. EV–ciliary membrane fusion or the triggering of signal transduction without EV internalization cannot be excluded. **E**, **F** Primary cilium made contact with another cilium of an adjacent cell, where N-glycosylated membrane proteins can mediate this interaction (**E**). The function of ciliary interconnections is poorly documented. In the hippocampal pyramidal neuron, a primary cilium may function as a postsynaptic site when it comes into contact with varicosities of serotonergic axons from the brainstem (**F**). Upon stimulation, serotonin/5-hydroxytryptamine (5-HT) released from synaptic vesicles can bind to primary cilia bearing G-protein-coupled 5-HT receptors, forming “axo-ciliary” synapses. **G**, **H** Interactions between the intertwined primary cilia are largely uncharacterized; whether they form continuous (**E**) or discontinuous (**G**) contact zones, enabling interactions of several cilia remains to be explored. EVs and/or other components (e.g. galectins) could initiate these ciliary interactions (**H**). Subdomains of primary cilia are indicated. The potential fate of a given action is indicated in italics. Items are not drawn to scale. DC, daughter centriole; MC, mother centriole; TGFβ, transforming growth factor-beta; PDGF, platelet-derived growth factor; GPCR, G protein-coupled receptor; mTOR, mammalian target of rapamycin; ECM, extracellular matrix. Illustrations are based on data and/or schemes presented in Refs [17, 38, 61, 73, 78–90]

The architecture of the cilia comprises several subdomains in addition to the axoneme, including the ciliary membrane, the ciliary tip, the transition zone, the ciliary pocket/periciliary membrane and the basal body (Fig. 1; see below Fig. 2) [31]. The transition zone at the base of the cilium acts as diffusion barrier that controls the entry and exit of ciliary proteins [32], while the basal body (derived from the mother centriole) serves as a nucleation site for axonemal growth that leads to cilium biogenesis once the cell leaves the cell cycle [33]. When the cell re-enters division, the cilium is resorbed and the basal body/mother centriole migrates to the perinuclear cytoplasm and organizes the centrosome, establishing a link between cell cycle and ciliogenesis [34]. Multiciliated cells generate hundreds of basal bodies through massive centriole amplification [35, 36]. The basic structure and organization of ciliary structures and their variations (primary or multiciliary, presence or absence of ciliary pocket) and ciliogenesis pathways in various cell types (e.g., mesenchymal/fibroblastic versus epithelial cells) are described in more detail elsewhere, as they are beyond the scope of this article [7, 37–41].

Several hundred proteins are implicated in the biogenesis and maintenance of ciliary structures. Exhaustive catalogues of the composition of various types of cilia, including primary cilia from different tissue sources, which could differ depending of the cell origin of cilia, were obtained by large-scale proteomics and protein interactomes [42–48] (reviewed in Ref [49]). In motile cilia, including longer flagella, motility is ensured by the coordinated activity of motor complexes consisting of outer and inner dynein arms [41]. They are organized in two rows with outer doublet microtubules allowing the ciliary bending and a wave-like motion (Fig. 1) [16]. In the nodal cilia, the motor complexes allow a movement in a circular, and often in a tilted fashion. Interestingly, in the immotile kinocilia the inner dynein arm complex is missing [29]. Motor proteins are also important for the non-vesicular intraflagellar transport (IFT). The kinesin and dynein motors enable anterograde and retrograde transport of cargo proteins into and out of the primary cilium. This mechanism is well described, as is the large heterooctameric coat complex, known as the Bardet-Biedl Syndrome complex (BBSome), which links IFT complexes to the ciliary membrane [50, 51]. Besides protein components, specific lipids such as sphingolipids and phosphoinositides, as well as cholesterol-rich membrane microdomains, are instrumental membrane organizers of ciliary signaling, structure and dynamics [52–54].

Mutated proteins causing dysfunction of cilia are responsible for disorders, collectively known as ciliopathies, that affect almost all tissue/organ systems. The targets of these mutations may be associated with ciliary structures or regulate them indirectly in terms of expression or assembly (reviewed in Refs [55–57]). Deciphering the causes of such phenotypes and the molecular players involved helped to understand the role(s) of the different ciliary compartments and the mechanism of the IFT that allows cargo trafficking and molecular signaling [58]. It is important to bear in mind that some ciliary proteins are found in sites other than the ciliary compartment and may be involved in functions unrelated to the primary cilia [59]. Discovery of new proteins that are either directly or indirectly involved in the organization of ciliary structures could reveal novel, unexpected roles for these ancestral organelles.

## Functions of the primary cilium

### The primary cilium as a sensory organelle

Although the primary cilium has been described for more than 100 years [60], its role has long been enigmatic, in contrast to the more obvious functions of motile cilia. It was thought to be functionally dispensable, a vestigial organelle. Given that the primary cilium is associated with non-dividing cells and must be disassembled prior to mitosis, a link with cell division has been proposed. The primary cilium has been considered as a sequester of the centriole, consequently regulating the cell cycle (reviewed in Ref [61]). Thus, it may serve as a structural checkpoint for re-entry into the cell cycle, and a link between the ciliary length and cell cycle progression has been proposed (reviewed in Ref [62]). Nevertheless, the exact timing of primary cilia resorption during the cell cycle depends on the cell type, while assembly can occur throughout the cell cycle, with the exception of mitosis [63] (reviewed in Refs [64, 65]).

As the primary cilium projects into the extracellular environment, it was also thought to act as a sensory structure, sensing and responding to external signals (e.g., mechanical-liquid flow, chemicals, light signal, osmolarity), thus participating in mechano-sensing signal transduction [61, 66–68]. This led to the demonstration that the primary cilium responds to certain mechanical signals, such as fluid shear stress, by generating an influx of extracellular calcium into the intracellular compartment, resulting in increased cytoplasmic calcium levels (Fig. 2A) [69–73]. This phenomenon is mediated by calcium channel protein complexes and was reported for various tissue and organ systems such as kidney, bone and blood vessels [74–76]. A physicochemical signal transduction takes place in the outer segment, a modified primary cilium, of the photoreceptor cells where light stimuli are converted into electrical impulses as part of the phototransduction cascade [77].

Several signaling pathways have been proposed to depend on the primary cilium, in which molecular players are embedded in the membrane, and respond to external biochemical cues such as morphogens and growth factors (Fig. 2B). For examples, primary cilia coordinate, among others, the Hedgehog, Wingless/int-1 (Wnt), Notch, transforming growth factor-beta, platelet-derived growth factor, and various G protein-coupled receptor (GPCR)-mediated signaling pathways that occur in a variety of cell types [73, 91–97]. Of note, there has been some debate regarding the role of ciliary structures in the canonical Wnt/β-catenin signaling [98, 99]. We refer readers to some compelling review articles that unravel the intricate relationship between primary cilia and Wnt pathways, as well as the impact of Wnt signaling on the regulation of cilium biogenesis and function [100, 101]. The activation and/or repression of the aforementioned signaling pathways can trigger a cascade of events that impact cell survival, proliferation, differentiation, and directional migration [102]. A functional interplay between primary cilia and the autophagy process has also been proposed, and the underlying mechanisms are being uncovered [103]. These topics and their mechanistic details are discussed in several excellent reviews [17, 34, 78, 94, 104, 105]. Overall, the primary cilium acts as a signaling platform during development, influencing stem and progenitor cell activities and/or tissue modeling, as well as homeostasis in adulthood.

### The primary cilium as a membrane source for ectosomes

#### Release of ciliary ectosomes

In addition to physicochemical signal perception, latest observations shed light on the role of primary cilia as communication hubs, emitting and docking extracellular vesicles (EVs). Indeed, the role of cilia as sources of donor membranes for the biogenesis of EVs is less well known. EVs are emerging as new communication devices enabling the cells of a given tissue or organ to have an impact on their immediate or distant surroundings [106, 107]. These extracellular entities are found in various body fluids and can be small (< 150 nm in diameter) or large (> 150 nm, up to 10 μm), and contain bioactive components such as nucleic acids, proteins and lipids that often reflect the biological conditions of the donor cells. In diseases such as cancer, they can contribute to the spread of oncogenes and alter the tissue microenvironment [108]. Various mechanisms have been described to explain their release [109]. In a nutshell, they can either originate from the endosomal system and be discharged as exosomes, or directly bud from the plasma membrane and be released as microvesicles/ectosomes [110]. The release of small ectosomes is called ectocytosis. Ectosomes can originate from various membrane protrusions [111, 112], including cilia, positioning them as novel sources of EVs [89, 113–117]. Ciliary ectosomes can be described as a special class of EVs (reviewed in Refs [81, 86, 87]). Their budding was observed at the ciliary tip, along the shaft or at the base/periciliary membrane (Fig. 2C) [11, 114, 118–121]. The last was observed in sensory neurons of *Caenorhabditis elegans* [122]. The composition of these subpopulations of ciliary EVs remains poorly described [84, 114, 119, 120, 123]. Nevertheless, the identification of specific biomarkers of ciliary ectosomes associated with a given body fluid could be informative about physiological conditions and diseases.

Mechanistically, EVs can be shed constitutively, as observed in the green alga *Chlamydomonas reinhardtii*, a unicellular flagellate, or in a signal-dependent fashion, following stimulation of GPCRs in mammalian primary cilia, when the retrograde transport mechanism is blocked [119]. Ciliary fission requires local (and transient) polymerization of G-actin to F-actin [12, 124, 125]. Various actin interactors and regulators such as myosin 6, drebrin, α-actinin 4 and actin-related protein 2/3 (Arp2/3) complex, and specific lipids such as phosphatidylinositol 4,5-bisphosphate and its regulators, phosphoinositide kinases and phosphatases, can participate in the ectosome release. Players involved in the ciliary disassembly such as Rho kinases Rac1 and Cdc42, Aurora kinase A and histone deacetylase 6 (HDAC6) can be also involved [46, 54, 119, 120, 126, 127] (see below). Besides molecular players and actin polymerization, distinct membrane microdomains, membrane curvature and tension might contribute to ectosome release [89, 124, 128, 129]. However, the mechanical details, including the sequential events leading to the budding, contractile ring formation and scission of ectosomes from ciliary structures, have yet to be established (reviewed in Ref [85]).

#### Functional impact of the release of ciliary ectosomes

The shedding of ciliary ectosomes can participate in the discharge of obsolete components impacting various signaling pathways, and potentially regulate the fate of the donor cell [105, 119]. In progenitor cells, as seen in the neuronal system, the release of the stem (cancer stem) cell biomarker prominin-1 (CD133) via ectosomes derived from the ciliary tip, among other sources, is apparently linked to the differentiation process [114]. Of note, the association of prominin-1, a cholesterol-binding glycoprotein, with numerous pathways (reviewed in Refs [130, 131]), including Hedgehog and Wnt signaling pathways, further strengthens its involvement in cilia-related functions, including those regulating cell cycle progression [101, 132–134]. Moreover, prominin-1 interacts with proteins that can influence the dynamics of primary cilia, including small regulatory ADP-ribosylation factor-like GTPase 13b (ARL13b), HDAC6, Arp2/3 complex and phosphoinositide 3-kinase [135–139]. This multitude of interactions has made prominin-1 an important player in signaling pathways associated with primary cilia as well as in the formation of ciliary structures and ectosomes derived thereof [114, 132, 137, 140–143] (reviewed in Refs [89, 131]).

Interestingly, some proteins are sorted into ciliary ectosomes due to a dysregulation in their signaling pathways like the somatostatin receptor 3. This GPCR accumulates after exposure to somatostatin at ciliary tips before being released extracellularly in association with ectosomes in mutant kidney cells lacking Arl6 [119]. The Arf-like GTPase Arl6, together with the BBSome complex and the conformational sensor β-arrestin 2, is involved in the classical pathway of endocytic retrieval of activated GPCRs from the ciliary membrane to the cell body [144, 145]. Similarly, ciliary ectocytosis of the GPCR GPR161, a negative regulator of Hedgehog, enables transduction of Hedgehog signaling when retrograde transport is disrupted in β-arrestin 2-defective cells [119]. The release of the aforementioned cellular proteins by ectocytosis was mainly observed at the ciliary tips or shafts. The release of EVs from the periciliary membrane or the ciliary pocket may also play a role in the removal of certain components, most likely in the discharge of excess of imported ciliary proteins, thereby regulating cilia composition as a safeguard mechanism [84, 122]. Therein, exosomes are likely discharged after fusion of the multivesicular body with the plasma membrane.

Ectosome release can also control the length of primary cilia and their disassembly prior to cell division [146, 147]. For example, the interaction of prominin-1 with HDAC6 may affect the release of ciliary ectosomes by influencing the level of alpha-tubulin acetylation in the axoneme [132], which in turn may trigger ciliary disassembly [148, 149]. In addition to the ectocytosis of small ectosomes, the upper part or the entire primary cilium can be eliminated by processes called decapitation and autotomy (cilium amputation), respectively, and both mechanisms could contribute to cell cycle-dependent disposal of the primary cilium [120, 150] (reviewed in Refs [85, 151]). In contrast, the retention of ectosomes can create membrane outgrowths as seen in the connecting cilium of photoreceptor cells, and thus regulate the integrity of the outer segment [152]. There, the release of ectosomes is suppressed by peripherin-2 and nascent membrane evaginations lead to the formation of new photoreceptor discs (reviewed in Refs [153, 154]. It should be noted that ectopic overexpression of prominin-1 in kidney cells results in primary cilia, albeit rarely, with multiple membrane outgrowths at their tips resulting in a flower-like morphology [89]. This may result from a defective EV release mechanism and resembles the early membrane evaginations observed in photoreceptor cells where prominin-1 is enriched and plays a role in organizing their structural alignment [141, 155] (reviewed in Refs [156, 157]).

It is worth mentioning that particular “subdomains” of the primary cilium that appear as bulges in the membrane or swelling entities along the ciliary shaft have been described [158]. These dynamic structures, known as ciliary bulbs or ciliary extracellular-like vesicles, have been observed in primary cilia of various cell types in fixed cells and/or tissues, as well as in living cells [158–160], ruling out the possibility that they are artifacts caused by chemical fixation. They might contain large enclosed vacuole-like structures [121, 161], or could delineate regions of the ciliary membrane that are sensitive to osmotic pressure [159]. Their movement along the cilium seems to be kinesin-dependent, and their formation can be inhibited by ceramide-based vesicle blockers [160]. Specific proteins were reported to be associated with ciliary bulbs, notably monosialodihexosylganglioside (GM_3_) synthase [158], which is coherent with the presence of GM_3_ in primary cilium [129]. CD63, a protein widely used as a marker of exosomes [162], was also found therein. The ciliary bulbs are stimulated by fluid shear stress in a GM_3_ synthase-dependent manner, and they can be released from the tips of cilia by the physical force of fluid movement [158, 160]. The exact function of such a transient structure remains to be established, but silencing its components, e.g., bone morphogenetic protein receptor type 2, junctional adhesion molecule A and progesterone receptor membrane component-2, causes distinct ciliary phenotypes such as the absence of the ciliary bulb or longer cilia. Physiologically, silencing of ciliary bulb components can result in cardiovascular dysfunctions as well as left-right asymmetry defects affecting cardiac looping, kidney cysts and curved body axis – phenotypes. These abnormalities are driven by the impairment of ciliary functions as observed in zebrafish and murine models [160].

In addition to modulating signaling pathways by expelling key ciliary components or the ciliary structure itself, the release of EVs from primary cilia may also contribute to intercellular communication. These ciliary ectosomes, detected in body fluids such as cerebrospinal fluid and urine, could facilitate the exchange of molecular signals between neighboring cells or over long distances, consistent with the established roles of EVs in systemic communication [88, 163]. Further studies are required to assess the physiological effects of the uptake of ciliary ectosomes by recipient cells.

Interestingly, the primary cilium may act not only as an ectosome-donor membrane, but also as a receptive structure (Fig. 2D) [118, 164, 165] (reviewed in Refs [86, 163]). After binding to the cilium, EVs (ectosomes or exosomes) may surf along the shaft to the ciliary base, where they can enter the cell by endocytosis, as observed with filopodia [38, 82, 166]. It is also possible that EVs fuse with the ciliary membrane or that the signal transduction might be transmitted without EV internalization [118]. These novel topics also require further attention as does the identification of the surface interactors that regulate the binding of EVs to the membrane of the primary cilium, and perhaps mediate their transport to the ciliary base (reviewed in Refs [89, 167]).

### The primary cilium as a mediator of cell-cell contact

Although primary cilia are typically considered solitary organelles, elongated cilia physically interact with those of neighboring cells [79, 159], forming connections that resemble closed-ended tunneling nanotubes (Fig. 2E) [168, 169]. Thus, several adjacent cells can interact with each other in this way [79]. Such interconnected primary cilia have been noted in in vitro cultures of kidney epithelial cell lines [159]. In human embryonic tissue samples, the cilium-cilium interaction was not only observed between neighboring epithelial cells, but also between cells on opposite sides of the embryonic pancreatic duct epithelium [170]. The connection of primary cilia has also been detected in the lumen of collecting ducts of the developing murine kidney [171]. As proposed by Nielsen and colleagues, cilia intertwining could be part of the sensory signaling machinery that regulates the initial development of the ductal system [170], where such cilium-cilium interactions could sense the size of the ductal lumen and, in some way, control cell proliferation. In line with this hypothesis, the number of such interactions is higher in the livers of 2-day-old mice than in those of 1-year-old animals [80].

In addition to the cilium-cilium interaction, long cilia can interact with the cell surface of neighboring cells [13, 80, 159]. When in contact with adjacent cells, primary cilia may function analogously to actin-dependent cytonemes and transmit signals in a paracrine manner [172], an aspect that warrants further investigation. It should be noted that, during neural development, cytonemes found in the lumen of the mouse ventral neural tube transport Sonic Hedgehog to apically-located progenitor cells via a direct contact with primary cilia, which again act as signaling hubs, thereby participating as receiving organelles in the action of morphogens [173]. This mode of intercellular communication may act sequentially, in conjunction with prominin-1^+^ ectosomes derived from neural progenitors [113, 114] (see above), to transmit the appropriate cell fate determination to adjacent responding cells during the embryonic brain development. Moreover, cilia can also establish a functional synapse-like interconnectivity as elegantly demonstrated for the interaction of primary cilia of rodent hippocampal pyramidal neurons with the neighboring varicosities of brainstem serotonergic axons that contain synaptic vesicles [174]. Upon axonal stimulation, serotonin is released onto cilia leading to a cellular response like an increase of RhoA activity via the non-canonical G_αq/11_ pathway (Fig. 2F). Such axo-ciliary synapses were also reported in human brain samples, i.e. between glial primary cilia and neuronal cells [175], and in the peripheral nervous system, where beta cell primary cilia contact cholinergic presynaptic terminals within pancreatic islets [13], suggesting a widespread phenomenon (reviewed in Ref [90]). These exciting novel aspects of ciliary structures acting as postsynaptic sites need further characterization.

Although we cannot rule out that cilium-cilium or other cilium-cell connections may be artifacts generated either during cell manipulation or processing, or during tissues preparation, the above-mentioned examples are nevertheless reminiscent of flagellum-flagellum contacts observed in *Chlamydomonas reinhardtii* during the mating process, a phenomenon promoted by adhesion glycoproteins referred to as agglutinins [176]. In line with this, Ott and colleagues demonstrated that cilium-cilium contacts observed in mammalian epithelial cells are promoted by adhesive force created by glycoproteins. They showed that these interactions are impeded by swainsonine, an inhibitor of mannosidase II that processes N-linked glycan moieties [80]. Moreover, the direct interaction between cilia is calcium-independent, suggesting, albeit indirectly, that cadherin or integrin molecules are not involved [80]. Interestingly, the same study reported that the cilium-cilium interaction is a dynamic two-step process, in which the tip of one cilium comes into contact with the shaft of another cilium in the immediate vicinity, leading to a zipper-like extension of the interacting zone, generating a long-lasting cilium-cilium connection [80]. Yet, the fusion of the two primary cilia was excluded [80]. Interestingly, a recent report has shown that Arl13/ARL13b regulates juxtaposed cilium-cilium elongation in a BBSome-dependent manner in sensory neurons of *Caenorhabditis elegans* [177]. Whether ARL13b interactors such as prominin-1 are involved in these processes will require further investigation. If prominin-1 is implicated here, it could play a similar role to that postulated for the organization/alignment of nascent membrane evaginations in photoreceptor cells (see above). The general characteristics of the direct interaction between ciliary structures remains to be explored in more detail, in particular whether firm continuous or discontinuous contact zones are formed between two (or three) cilia (Fig. 2G). A role for EVs (or other components, e.g., galectins) in initiating cilia interactions, and thus complementing the action of ciliary membrane glycoproteins in the formation of the ciliary interconnection (Fig. 2H), is not ruled out [177].

The functions of the ciliary interconnection linking distinct cells, beyond serving as a specialized platform for the exchange of signaling molecules or facilitating mutual sensing and synchronized responses to external stimuli remain to be elucidated. Whether ciliary interconnections are involved in the reorganization of plasma membrane lipids and/or proteins at the contact zone between connected cilia and have an impact on ectosome release (or other unidentified processes) also needs to be determined. It is noteworthy that the contact zone(s) between primary cilia may have distinct compositions depending on the cells of origin and/or may perform different functions. A subset of axo-ciliary synapses is thought to contain gap junctions [175]. These new features of the primary cilium could explain why the organization of the axoneme (9 + 0, see above) is not regular throughout the length of the cilium, thereby potentially providing structural flexibility (and/or deformability) notably at the ciliary tip to accomplish their newly described features, i.e. when they come into contact with other structures, or during the release of ectosomes.

Besides interactions with other membranous structures, i.e. cilia, cytonemes, nerve cell axons and cell bodies or EVs, primary cilia can also bind to the extracellular matrix (ECM) as demonstrated in chondrocytes and vascular smooth muscle cells [178, 179]. ECM receptors, such as certain integrins and neural/glial antigen 2 proteoglycan, have been shown to localize to primary cilia [178]. In this context, it has been proposed that ciliary structures can detect mechanical and physicochemical alterations in the pericellular environment [178]. Moreover, the cilium-ECM interaction could promote cell migration, as suggested by a scratch-wound assay arguing for its potential importance during development or after tissue damage [179].

Overall, recent studies in various fields of research have highlighted new roles for primary cilia. These signaling hubs protruding into the extracellular environment could mediate or support tissue and organ development and adult homeostasis, and/or play a role after injury. Since morphological and functional alterations of primary cilia cause various disorders and diseases analyzing defects related to proteins involved in ciliary assembly, function and communication could significantly advance our understanding in a broad spectrum of developmental and health anomalies.

## Concluding remarks and future directions

The primary cilium, an organelle that has been known for a century, advances to a next level of understanding in relation to its hitherto overlooked functional roles in signal transduction and intercellular communication. Primary cilia, in addition to their well-established mechano- and chemosensory roles as well as in photoreception via the modified sensory cilia of photoreceptor outer segments, are increasingly recognized as hubs in intercellular signaling at several levels. Communication can take place over a short or long range via EVs and/or soluble factors, with cilia functioning as both EV donor and recipient organelles, or over a short range via physical contacts such as cilium-cilium, cilium-cytoneme and cilium/axon interactions. The study of these new cellular facets of primary cilia should lead to a better understanding of intercellular communication, and perhaps new function(s). The identification of primary cilium surfactomes under various cellular conditions (physiological or pathological) and the associated molecular pathways could shed light on novel features that may be involved in tissue development and homeostasis, as well as in certain diseases when their actions are deregulated. These efforts would broaden our knowledge of fundamental ciliary processes and could be useful in promoting tissue engineering and as a diagnostic tool for classical ciliopathies and other, as yet less explored, conditions including diabetes and neuropsychiatric diseases.

## Data Availability

No datasets were generated or analysed during the current study.

## Abbreviations

Arp2/3: Actin-related protein 2/3
ARL13b: ADP-ribosylation factor-like GTPase 13b
BBSome: Bardet-Biedl syndrome complex
ECM: Extracellular matrix
EV: Extracellular vesicle
GM3: Monosialodihexosylganglioside
GPCR: G protein-coupled receptor
HDAC6: Histone deacetylase 6
5-HT: 5-hydroxytryptamine
IFT: Intraflagellar transport
Wnt: Wingless/int-1
