# Correction: The primary cilium as a multifunctional organelle: emerging roles and unanswered questions

**DOI:** 10.1186/s12964-025-02500-9

**Published:** 2025-11-13

**Authors:** Denis Corbeil, Kristina Thamm, Jana Karbanová, Christine A. Fargeas, József Jászai

**Affiliations:** 1 Biotechnology Center (BIOTEC) and Center for Molecular and Cellular Bioengineering, Technische Universität Dresden, Tatzberg 47-49, Dresden, 01307 Germany; 2 Tissue Engineering Laboratories, Medizinische Fakultät der Technischen Universität Dresden, Fetscherstr. 74, Dresden, 01307 Germany; 3 Institute of Anatomy, Medizinische Fakultät der Technischen Universität Dresden, Fiedlerstr. 42, Dresden, 01307 Germany; 4 Tissue Engineering Laboratories, Biotechnology Center, Technische Universität Dresden, Tatzberg 47-49, Dresden, 01307 Germany; 5 BIONCaRT GmbH, Fürstenweg 8, Grimma, D-04668 Germany

**Correction: Cell Communication and Signaling 23, 406 (2025)**

**https://doi.org/10.1186/s12964-025-02403-9**

Following publication of the original article [1], Authors would like to correct the online version of the figures as both figures are not visible.

The original article [1] has been corrected.

## References

[1] Corbeil D, Thamm K, Karbanová J, et al. The primary cilium as a multifunctional organelle: emerging roles and unanswered questions. Cell Commun Signal. 2025;23:406. 10.1186/s12964-025-02403-9. 41039495 10.1186/s12964-025-02403-9 PMC12490143

